# A QR code-enabled framework for fast biomedical image processing in medical diagnosis using deep learning

**DOI:** 10.1186/s12880-024-01351-z

**Published:** 2024-08-01

**Authors:** Arwa Mashat

**Affiliations:** Faculty of Computing & Information Technology, King Abdulaziz University, P. O. Box 344, 21911 Rabigh, Saudi Arabia

**Keywords:** Deep learning, Medical imaging, Decision making, QR code, Database, Diagnosis

## Abstract

In the realm of disease prognosis and diagnosis, a plethora of medical images are utilized. These images are typically stored either within the local on-premises servers of healthcare providers or within cloud storage infrastructures. However, this conventional storage approach often incurs high infrastructure costs and results in sluggish information retrieval, ultimately leading to delays in diagnosis and consequential wastage of valuable time for patients. The methodology proposed in this paper offers a pioneering solution to expedite the diagnosis of medical conditions while simultaneously reducing infrastructure costs associated with data storage. Through this study, a high-speed biomedical image processing approach is designed to facilitate rapid prognosis and diagnosis. The proposed framework includes Deep learning QR code technique using an optimized database design aimed at alleviating the burden of intensive on-premises database requirements. The work includes medical dataset from Crawford Image and Data Archive and Duke CIVM for evaluating the proposed work suing different performance metrics, The work has also been compared from the previous research further enhancing the system's efficiency. By providing healthcare providers with high-speed access to medical records, this system enables swift retrieval of comprehensive patient details, thereby improving accuracy in diagnosis and supporting informed decision-making.

## Introduction

In this scenario of model healthcare, the most important factor for patients and healthcare providers is the timely and accurate diagnosis of medical conditions. This stands as a critical measure of both patient satisfaction and also success of ensuring good health of the care-receiver. Yet, despite significant advancements in medical imaging technologies, delays in diagnosis still persist.

According to a study by Society to Improve Diagnosis in Medicine (SIDM), it has been reported that diagnostic delays affect an estimated 12 million American citizens annually, this further translates to 10% of the total patient deaths in the United States of America [1]. Further, according to another study conducted by Paramasivam et al. in 2017, it was found that diagnostic delay in patients of Tuberculosis (TB) in the state of Kerala was the primary reason for increased period of infectivity and increased severity of the disease in patients [2]. From a study conducted by Suneja et al. [3], it was found that primary reasons for diagnostic delay was due to – limitations in testing, unusual clinical presentations, and failure to collaborate with other specialists.

These delays that occur in the diagnosis not only impact patient outcomes but also incur substantial cost to both the health care provider and health care giver. In a study by Newman et al. [4] in the journal of Quality and Safety, it was estimated that diagnostic errors account for up to USD 100 billion in annual healthcare spending in the United States of America alone. Furthermore, delays in diagnosis have been shown as a leading cause for malpractice claims as well.

The above issues further emphasize on the need for an innovative and cutting-edge technology driven solution that solves the problems of delay in diagnosis and also reduce the time and cost incurred for storage of medical data.

In order to handle these challenges, this paper proposes a novel methodology aimed at both accelerating medical diagnosis using first level diagnosis through deep learning & image processing methods and also by making the medical records more easily accessible for health care provider & receiver. In this endeavor data driven modelling of medical images is used and also using advanced database design methodologies to handle infrastructure for data storage.

In short, the paper shows a novel methodology towards accelerating the diagnosis of medical conditions while concurrently addressing the cost and efficiency issues associated with traditional data storage infrastructures. This approach centers on the development of a high-speed biomedical image processing system, augmented by machine learning algorithms, to streamline the diagnostic workflow and enhance the accuracy of clinical decision-making.

Statistical studies conducted by the John Hopkins Medicine [5] reveal that an estimated number of 80,000 to 100,000 deaths are due to errors during medical diagnosis, this makes the issue a a major public health concern. Furthermore, it is also to be noted that delays in diagnosis have been linked to increased morbidity and mortality across various medical specialties, ranging from oncology to emergency medicine.

In order to mitigate the above-mentioned risks and improve patient outcomes, this proposed approach integrates machine learning techniques, such as semi supervised deep learning model and feature reduction techniques, into the diagnostic process. By leveraging the vast amounts of medical imaging data available online on free-issue repositories, these algorithms can identify patterns and anomalies that may not be readily apparent to human observers, thereby, providing the healthcare providers with a first level observer results on the diagnosis – thereby reducing the time of diagnosis.

The advanced image processing techniques, including Singular Value Decomposition (SVD) and QR coding are taken, to speed-up & optimize medical image retrieval and analysis [6]. This shall enable healthcare professionals to expedite the analysis of images and make timely and informed decisions, ultimately reducing the time-to-diagnosis and improving patient outcomes.

The speed of medical diagnosis by employing statistical processes along with machine learning driven methodologies are considered, the need to speed up biomedical image processing to help health care givers provide better treatments is emphasized.

For example, in a study conducted by researchers at Stanford University [7], it was shown that deep learning algorithms were employed to analyze X-ray images for diagnosis of pneumonia. It was observed that the algorithm showed a level of accuracy comparable to expert radiologists. Similarly, a study published in Nature Medicine demonstrated [8] the efficacy of deep learning in diagnosing skin cancer from medical images, achieving a classification accuracy exceeding that of dermatologists.

The main objective is to address the issue of delayed detection and also, poor storage frameworks in the health sector through using machine leaning plus improved image processing. The proposed methods could work in real practice and their abilities to change the diagnosis and treatments of various ailments thereby reducing preventable death cases and enhancing better health care globally.

In this paper, the following contributions are:

- To propose a novel framework for storing medical images to reduce IT infrastructure cost of Health Institutions (Hospitals, Medical Colleges, or R&D centers) using cutting edge database methodologies such as SVD etc.
- To develop novel algorithms using Deep Learning to develop a first level study layer on diagnoses made using biomedical image processing.
- To evaluate the proposed method with other deep learning-based methods and database design philosophies implemented and generate comparative analysis of the model.
- To assess the effectiveness of the work using metrics and visualization tools.

The structure of the paper is as follows: The Introduction is covered in Sect. “ Introduction”, the Prior Research in the Field is highlighted in Sect. “ Literature review”, the Detailed Proposed Framework and Methodology is discussed in Sect. “ Proposed methodology”, the Results are evaluated and discussed in Sect. “ Experimental results and discussions”, and possible Future Directions are discussed in the last section.

## Literature review

According to Wen. W et al. [9], the loss or unrecoverability of a secret key is a difficulty for medical picture content protection and security sharing when using vanilla blockchain technology. Consequently, their research offers a blockchain-based, integrated tiny shadow QR code-based verified medical image-sharing approach. Initially, a little shadow picture is created by the use of a covert image-sharing technique founded on the Chinese remainder theorem. After that, a QR code with error correction integrated into it uses the shadow picture. This removes the chance that the secret key will be misplaced and unrecoverable in addition to safeguarding the security and integrity of the shadow image transmission over the public channel. The methodology in the study employs smart contracts to authenticate and restore secret pictures, hence reducing local demand, in contrast to other well-established ways. It also uses a hash bit stream for authentication. Additionally, an examination of the authors' suggested model reveals that the cover QR code is more secure and durable, and the retrieved hidden medical picture is lossless. This method is suitable for safe sharing and content protection in medical imaging.

According to Tao et al. [10], deep learning-based image fusion techniques have been a popular area of research in computer vision in recent years. These tactics are looked at in this study from five different angles. Initially, the advantages and theory of deep learning-based image fusion techniques are examined. Second, there are two main summary points for the image fusion approaches: Based on the different duties of deep learning in the feature processing stage, end-to-end and non-end-to-end image fusion approaches are divided into two categories: deep learning for feature extraction and deep learning for decision mapping. Depending on the kind of network, end-to-end picture fusion techniques are divided into three categories: encoder-decoder, generative adversarial, and convolutional neural networks. Third, the approach and data set for the use of deep learning-based image fusion techniques in the medical imaging field are summed up in two words. Fifth, the main challenges in medical picture fusion are analysed in two sections: data sets and fusion algorithms. Fourth, assessment criteria often used in the area are categorised into 14 components. Additionally, the tendency of future development is expected. This study offers a thorough review of deep learning-based image fusion algorithms, which is beneficial for providing a direction for further research into multimodal medical images.

Further elaboration was provided by Kumari et al. [11], who said that the rapid advancement of deep learning has significantly advanced the area of medical image analysis. Despite these successes, a significant obstacle to further developing deep learning models for medical image processing is the lack of large, well-annotated datasets. The development of data-efficient deep learning techniques has received more attention in recent years as a means of overcoming this obstacle. An extensive evaluation of data-efficient deep learning techniques for medical image interpretation is presented in this article. In order to do this, the methods are categorized based on the level of supervision they necessitate, including groups such those that require no supervision, precise supervision, partial monitoring, erroneous supervision, and little supervision..

Based on the literature review, the previous research works which employ QR, Deep Learning methods and Biomedical imaging for increasing the accuracy and speed of diagnosis, the emphasis towards employing solution towards optimizing the accessibility of information on-the-go is needed. None of the available works done earlier have done any activity towards handling the data storage, data security and transmission cases. The data is kept in a centralized fashion, which makes the data prone to cyberattacks. In addition to this, the deep learning models used in the studies are either supervised models or unsupervised models, the problems this pose is the system undergoes high number of iterations without much value to the overall system. This creates a heavy load on the system with no apparent qualitative output.

This paper uses features of Deep Learning & Image processing methodologies to handle these shortcomings of earlier methodologies, resulting in a ground-breaking approach to cyberbully identification that maintains the highest level of user information privacy and data security.

Primarily, the gaps found in the available literature reviews points towards lack of attention towards speed of data access, optimization of storage of data and Table 1 present the comprehensive analysis of previous work.

**Table 1:** Comparative study of systems proposed in earlier works

#	Paper title & Ref No	Advantages	Disadvantages	Techniques Used	Dataset	Accuracy
1	**Wen et. al** [9]	**High data security based on Blockchain**	**High run time leading to computational expensive system**	**QR Codes + Blockchain**	**IDA**	**97.12%**
2	**Tao et. al ** [10]	**Speed of Image retrieval significant**	**Loss of key feature characteristics**	**Image fusion technology**	**Github + Kaggle**	**97.34%**
3	**Tao et. al ** [10]	**Image recognition with high accuracy**	**No data security steps taken**	**Data breach issues**	**VISR Dataset**	**97.29%**
4	**Kumari et. al ** [11]	**Connected image analysis with high recall for “same agent” mapping**	**Low speed of information retrieval**	**Multi modal deep learning**	**IDA**	**96%**

## Proposed methodology

This paper envisages novel a method which is coined as QR driven high speed Deep Learning Model (Q-DL) methodology to handle develop a high-speed image processing system with QR ingrained in the process with an optimized data based design for efficient storage of information.

In the approach defined in this study, a semi-supervised deep learning model, such as t-SNE, along with image processing tools is employed with to improve on the statistical parameters of the model, speed of diagnosis, efficient data storage and privacy of the process.

It is to be noted that the t-SNE model used in this paper also is used with some modifications to increase the accuracy and decrease computational time. In the particular t-SNE module, the modification that is done is during the classification processing, instead of going ahead with the traditional approach, the Bhattacharya distance is used between the features on the sub-space for similarity mapping during image processing and diagnosis process. The same methodology is also used during data base design based on image similarity, where concepts of Bregman Divergance to store information on a similarity space is taken. It is once again to be noted that the inspiration of using a similarity space for image processing have been derived from a study made by Liu et. al [12], who use the Mahalanobis distance based similarity mapping approach for fault detection in civil structures.

In the proposed model, the information based on similarity mapping to handle scenarios which require high speed transactions is truncated. GAN is specifically used to measure the performance of the proposed Q-DL method with respect to various statistical results, due to the prevalence of GAN models in modern day deep learning models across all fields.

A semi-supervised model leverages the small amount of labeled data along with a larger pool of unlabeled data to achieve high accuracy and robustness in the learning process.

In this way, the model achieves faster run time due to actively replacing information in the stack, that is similar to the other information available with details of reconstruction stored as a part of the QR code [13–22]. In addition to that, the use of a semi supervised model also ensures that unnecessary power of compute is not burnt to handle information and processes which have no relevance in the model.

**Fig. 1:**
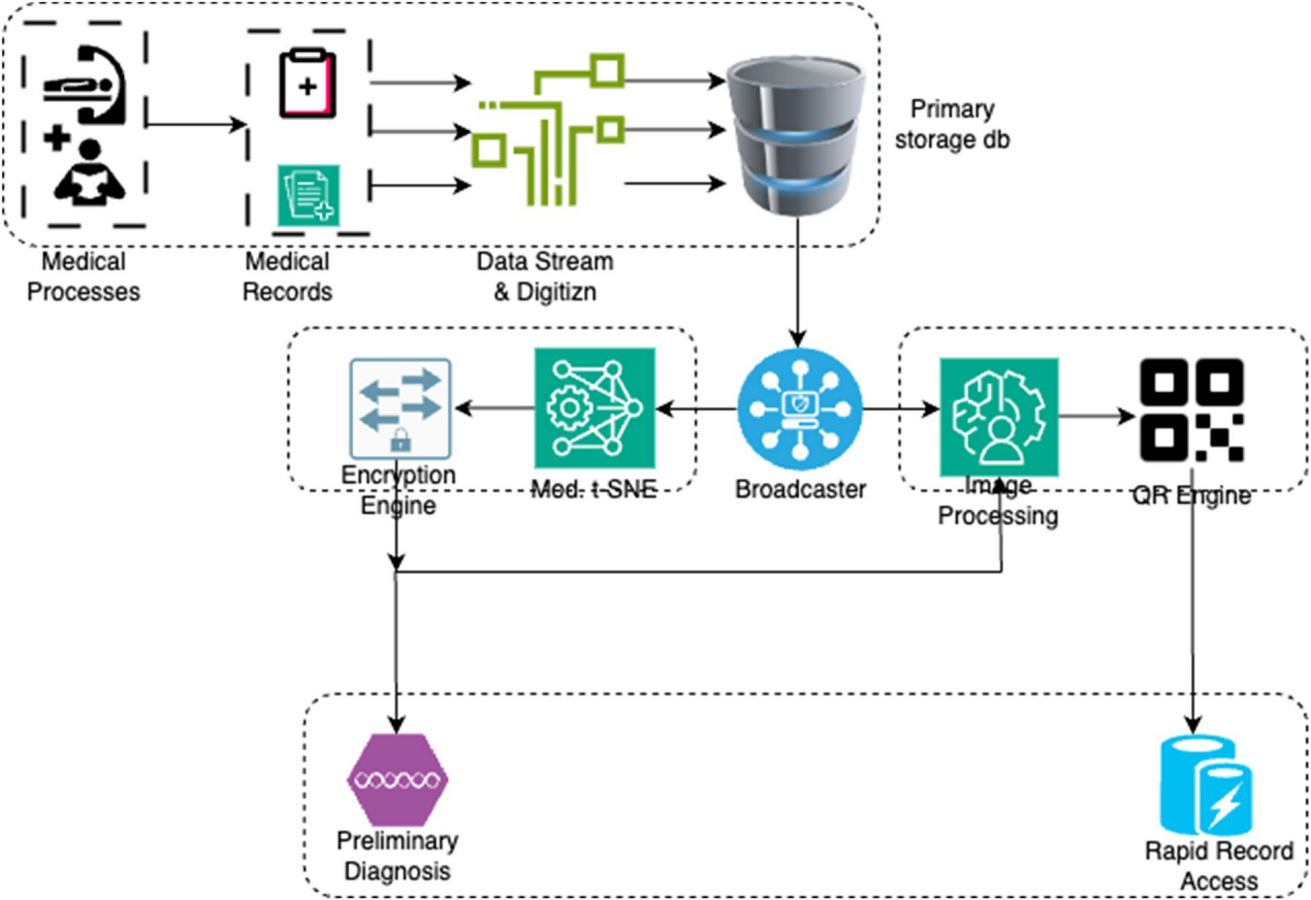
Overall structure of the Q-DL model

The architecture given in Fig. 1 shows the complete data flow and working of the proposed design:

The data is prepared to be ingested into the model in order to achieve the intended performance. It eliminates unintended consequences, avoids problems, and boosts dependability in the signal. The “Q-DL” dataset, which is the stage dataset, is used for activities including data cleaning, data normalisation, and data stream construction.

In order to prevent high level outliers from having any form of impact on the model, all blank value fields and social media comments with unclear word stems are removed from the database.

Additionally, this technique minimizes the run duration of the model by executing features to eliminate the impacts provided in the dissimilar scale [15]. The min–max normalization procedure is a strategy that data scientists utilize frequently in their work. Since this approach is more akin to feature scaling, it has drawbacks of its own. Specifically, the normalization greatly reduces the model's bias. As a result, this model makes use of a relatively unexplored normalization approach that connects the dataset with the standard deviation found in the dataset.

The data is scaled in accordance with the specifications of the suggested model by normalizing it using the Z-Score normalization process.

1 $${x}^{\#}=\frac{x- \overline{x}}{\sigma }$$

This is the Z-normalized value, denoted by ${x}^{\#}$. The data's average value, or mean, is represented by $\overline{x }$, and its standard deviation is denoted by σ. For all numerical data, this normalization is applied immediately; for non-numeric data, a one-hot encoding or normal encoding procedure is first performed on the data [16].

Subsequently, is the step where the physical data is stored in digitized form in a primary storage database for further processing. Real-time processing is made possible by Event-Driven Architecture, which is essential for gathering and managing data streams in real time.

In order to create networked applications, representational state transfer APIs adhere to a set of architectural principles that offer a standardised means of system communication [17]. Webhooks improve the responsiveness of API integrations by enabling real-time communication across systems by setting off events in one system depending on actions or modifications in another. With regards to concurrency, it is to be noted that as per Fig. 1 in the paper, a data broadcaster and data streaming end points are used. These services shall allow concurrence upto 20, as a base. The same can be increased with more robust hardware, if needed.

In the proposed methodology where the speed of result from the solution is of paramount importance, the role of state transfer APIs are even more to ensure the efficacy of data transfer as well as completeness of data pushed from one node to another.

Later, a data broadcaster is developed which pushes the information to the deep learning and image processing units of the proposed design. In the proposed architecture, the data broadcaster serves as the primary system for transmission of information to both the image processing and deep learning units. It operates using well-known and robust protocols like TCP/IP or MQTT, ensuring efficient and complete data transfer.

Prior to transmission, the broadcaster may pre-process the data, applying techniques such as normalization, scaling, or feature extraction to optimize its suitability for downstream processing tasks.

**Fig. 2:**
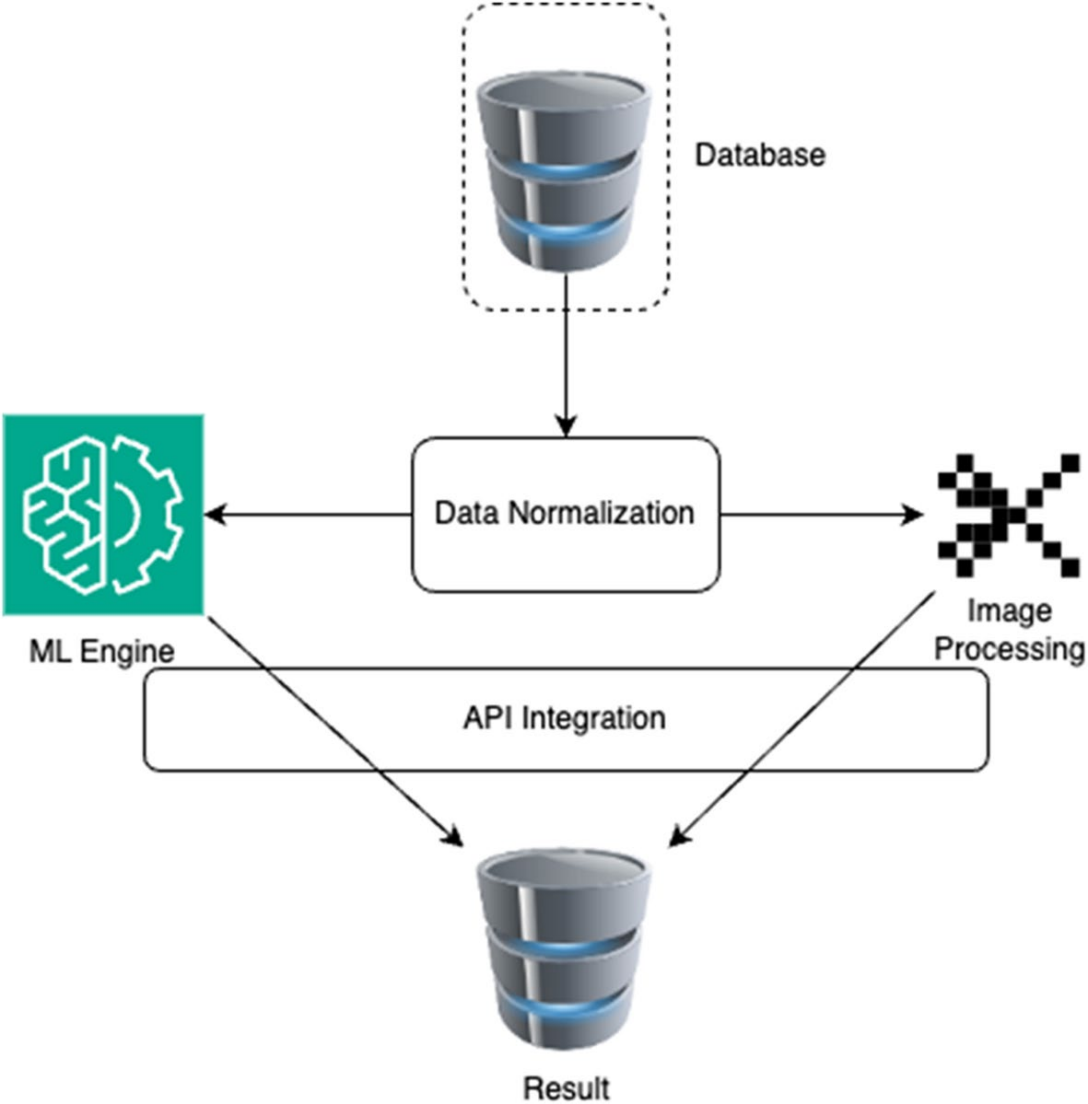
Internal methodology of the process flow

The above process is defined in the below Fig. 2:

The deep learning engine used in this model uses a semi supervised model. The model used is a modified t-SNE algorithm, where concepts of similarity mapping are used—Bregman Divergence such as Bhattacharya Distance to reduce the number of features without particularly impacting the quality of result generated.

In this research methodology, the utilization of the t-SNE algorithm and the integration of Bhattacharya Distance within feature reduction hold pivotal roles, each contributing to the effectiveness of this approach [23].

An innate quality of Bhattacharya Distance is, it can reduce the computational time taken from similarity mapping by temporarily removing repetitive features, without significantly affecting the performance or behaviour of the model. Therefore, in the proposed model Bhattacharya Distance has been sued instead of classical Euclidean Distance methods to reduce Bregman Divergence.

t-SNE, or t-distributed stochastic neighbour embedding, is selected for its remarkable ability to transform high-dimensional data into lower-dimensional representations while preserving local and global structures. This is crucial in this context as it allows for the visualization of complex data patterns in a more understandable format, facilitating the identification of underlying relationships and clusters within the data. By leveraging t-SNE, inorder to enhance the interpretability and analysis of proposed deep learning model's output, ultimately aiding in the extraction of meaningful insights from the data.

Furthermore, the incorporation of Bhattacharya Distance within thus feature reduction process serves to optimize the efficiency of the deep learning engine. Bhattacharya Distance, a measure of the similarity between probability distributions, enables us to quantify the difference between feature sets while retaining critical information pertinent to the task at hand. By employing this metric, the dimensionality of this feature space is reduced without significant loss of discriminative power [18, 19]. This is particularly advantageous in scenarios where the original feature set is high-dimensional and contains redundant or irrelevant information, as it allows for more streamlined processing and improved model performance.

Traditionally, the tSNE working theory with all parameters is as follows:

1. Input: Dataset $X$ containing $n$ data points in $d$-dimensional space.
2. Output: High-dimensional data matrix *X*.
3. Compute the pairwise Euclidean distances *D* between all data points in *X*.
4. Apply a Gaussian kernel to *D* to obtain pairwise similarities *Pij*​ for each data point pair *i* and *j*.
5. Normalize the similarities *Pij*​ to obtain conditional probabilities *P(j*∣*i)*​ representing the likelihood of observing data point *j* given data point *i*.
6. Set the perplexity *P(erp)*, determining the effective number of neighbours for each data point.
7. Compute *P(j*∣*i)*​ such that the perplexity of *P(j*∣*i)*​ for each *i* is approximately equal to *P(erp)*.
8. Initialize the low-dimensional embeddings *Y* for each data point randomly or using another dimensionality reduction technique like PCA.
9. Compute pairwise Euclidean distances *dij*​ between embedded points *Yi*​ and *Yj*​.
10. Apply a Gaussian kernel to *dij*​ to obtain low-dimensional pairwise similarities *Qij*​.
11. Normalize the similarities *Qij*​ to obtain conditional probabilities *Q(j*∣*i)*​ in the low-dimensional space.
12. Adjust *Y* iteratively to minimize the Kullback–Leibler divergence *KL*(*P*∣∣*Q*) between the high-dimensional and low-dimensional distributions.
13. Define a cost function *C* as the KL divergence between *P* and *Q*.
14. Use gradient descent or another optimization technique to minimize *C* with respect to *Y*.
15. Iterate steps 5–7 until convergence, ensuring that *Y* stabilizes and adequately captures the data structure.
16. Once convergence is achieved, the resulting embeddings *Y* represent the lower-dimensional representation of the original data points.

The steps mentioned above is the process of a traditional t-SNE algorithm, however, in this processes the KL divergence with Bregman Divergence is replaced, and the Euclidean Distance with Bhattacharya Distance is replaced. Using this, a similarity mapping is imple meneted to reduce computational timing.

The modified t-SNE algorithm used in the proposed approach is as follows:

1. Input: Dataset $X$ containing $n$ data points in $d$-dimensional space.
2. Output: High-dimensional data matrix *X*.
3. Compute the pairwise Bhattacharya distances *Db* between all data points in *X*.
4. Set the perplexity *P(erp)*, determining the effective number of neighbours for each data point.
5. Compute *P(j*∣*i)*​ such that the perplexity of *P(j*∣*i)*​ for each *i* is approximately equal to *P(erp)*.
6. Initialize the low-dimensional embeddings *Y* for each data point randomly or using another dimensionality reduction technique like PCA.
7. Compute pairwise Bhattacharya distances *Dbij*​ between embedded points *Yi*​ and *Yj*​.
8. Adjust *Y* iteratively to minimize the Bregman Divergence *BG*(*P*∣∣*Q*) between the high-dimensional and low-dimensional distributions.
9. Define a cost function *C* as the Bregman divergence between *P* and *Q*.
10. Use gradient descent or another optimization technique to minimize *C* with respect to *Y*.
11. Iterate steps 4–8 until convergence, ensuring that *Y* stabilizes and adequately captures the data structure.
12. Once convergence is achieved, the resulting embeddings *Y* represent the lower-dimensional representation of the original data points.

Since, Bhattacharya distance is used, which has the internal feature of normalization of all input vectors, therefore, the computational time needed for kernelization of the data and normalization is saved in this process.

Mathematically, Bhattacharya distance is defined as the following,

Define, $P$ and $Q$ as two probability distributions over the sample space $S$. Therefore the Bhattacharya distance is defined as,

$$D\left(P,Q\right)=-\text{ln}({\sum }_{x \epsilon S}\sqrt{P\left(x\right)Q(x)} )$$

While the above formation works well for this approach, it is to be noted that this formula fits only for datasets or sample spaces which are discrete in nature.

For sample spaces which are continuous, a simple modification in the mathematical formula is done by replacing piecewise summation with a area integration as follows,

$$`D\left(P,Q\right)=-\text{ln}(\int\sqrt{P\left(x\right)Q(x)})$$

In addition to the traditional advantages of the Bhattacharya Distance, this modification is also beneficial to this approach for the following advantages,

When compared to other distance metrics, the Bhattacharyya distance might have varying levels of computational complexity. In contrast to more straightforward distance measurements like Euclidean distance, Bhattacharyya distance may need computations like square roots and logarithms, which might be computationally demanding. Therefore, whether working with huge datasets or real-time processing, the computational cost of Bhattacharyya distance computations may have an impact on the system's performance.

Data format may also have an impact on Bhattacharyya distance computation performance. Computing Bhattacharyya distance could be easier if the data are in a format that naturally fits probability distributions or if probability distributions are easily accessible. Nevertheless, this extra step may slow down the system as a whole if the data needs to be pre-processed or transformed in order to be represented as probability distributions. When there are complicated and nonlinear correlations between data points in high-dimensional spaces, Bhattacharyya distance can be especially useful. When compared to other, less complex distance measures, Bhattacharyya distance can offer a more precise indicator of the similarity of probability distributions in certain situations. However, the complexity of the data increases the computing cost of calculating Bhattacharyya distance, which might affect system performance.

The suggested approach handles cases that require for high-speed transactions by truncating the data based on similarity mapping. Characteristics that were not utilised in the model's construction and analysis have likewise shown data loss via similarity mapping, there is no need to give this particular issue special consideration while implementing the model.

### QR Generation:

QR code generation used in this approach is designed with the following factors in mind,

1. QR Code is faster than the baseline time complexity of the process – the baseline time complexity of QR code generation of information with 300 character is about 3 s.
2. QR Codes should be dynamic in nature, information coming in into the QR Engine should be for similarity, and in cases where more than 70% similarity is seen – QR tagging to be done rather than a complete new QR code generation.
3. QR Codes should be saved in a compressed form – compression is done using QR decomposition process and SVD to reduce loss of information in the process.
4. These QR codes will be available for immediate download via Kinesis data streams over 3 AZs globally.

Keeping the above in mind, the steps and processes involved in QR code generation is as follows [24]:

1. Data Encoding:
  Transform the supplied data into a binary format that may be used to encode QR codes. Depending on what kind of data has to be encoded, use encoding systems like byte, kanji, alphanumeric, or numeric encoding. As specified by the QR code standard, add mode and character count indications.
2. Error Correction Coding:
  After the data has been encoded, divide it into blocks and provide each block error correction coding. In order to provide redundancy to the data and facilitate error detection and repair, QR codes commonly include Reed-Solomon error correction codes. Using the QR code version and error correction level as a foundation, calculate the total number of codewords with error corrections.
3. Matrix Representation:
  Create a matrix format with the encoded and error-corrected data arranged in it. Based on the volume of data and degree of error correction, choose the QR code matrix's size and version. Put the error correction codewords and encoded data in the appropriate places inside the matrix.
4. Masking:
  Enhance the readability and scanning reliability of the QR code matrix by using a masking pattern. To change some of the matrix's modules' colour from black to white, select from a number of preset masking schemes. Analyse each masking pattern's penalty score, then choose the one with the lowest penalty.
5. Format Information and Version Information:
  To define characteristics like error correction level, masking pattern utilised, and QR code version, embed format and version information within the QR code matrix. Use predetermined formats to encode the version and format information, then insert them into designated spots inside the matrix.
6. Quiet Zone:
  To guarantee that scanning devices correctly identify and decode the QR code matrix, provide a silent zone around it. The white region that surrounds the QR code matrix is known as the silent zone, and it serves to shield it from outside disturbance.

**Fig. 3:**
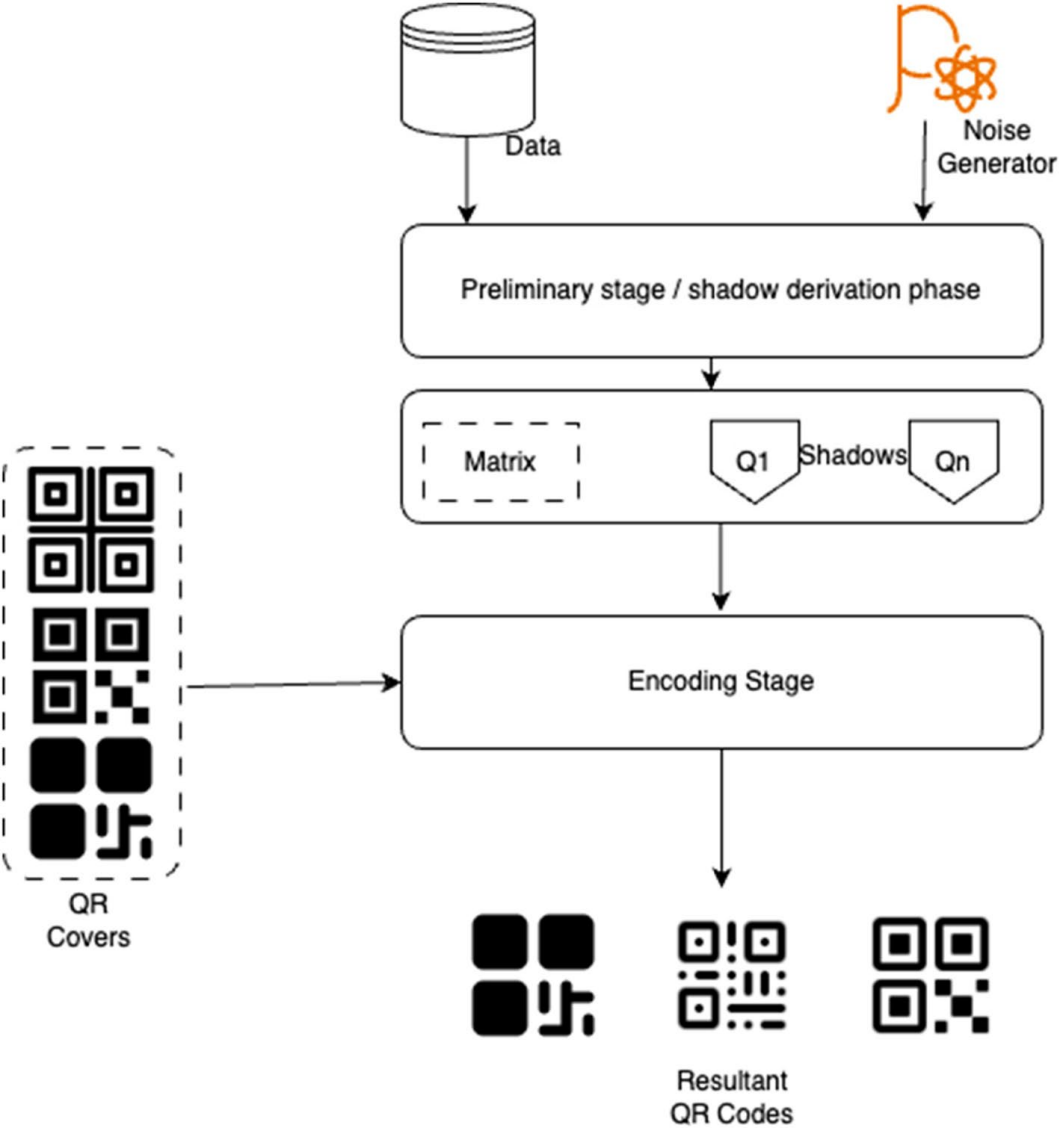
QR Generation process

The over process of QR Code generation is given below in Fig. 3:

From the above process,the basis of QR generation is the base data, noise and QR covers [25, 26]. These are the steps, where tSNE is used to speed up the overall process and make QR generation time & space efficient.

It is to be noted that, the problem of additional time and computational complexity given rise by generation of various QR codes is handled by using a fixed set of shadow QR codes as QR covers. In addition to this, the data is transformed to smaller and simpler forms using a modified t-SNE algorithm to make the process faster.

To ensure that the use of the same set of QR maps does not impact the solution’s innate performance, the authors have implemented harr cascde based QR reading process, where even the minutest changes can be picked up, therefore reducing the chance of confusion while model running.

### Proposed model algorithm with parametric tuning

First, pre-processing is done on medical photos to minimise noise and increase quality. To get the best possible image quality, methods including contrast augmentation and denoising are used. Image segmentation is used to identify areas of interest within the medical pictures after pre-processing. In this stage, structures or anomalies like tumours or organs are segmented using region growing or thresholding algorithms. Relevant characteristics are retrieved from the segmented pictures to reflect the underlying data. This entails examining the subdivided areas' texture, shape, and intensity-based features. The improved t-SNE algorithm receives these extracted characteristics as input.

The retrieved features are then subjected to the modified t-SNE algorithm. Bhattacharya distances or other similarity metrics appropriate for medical picture analysis are used into this approach. Reducing the feature space's dimensionality while maintaining the data's local and global organisation is the goal. This makes it possible to analyse and visualise the data in a meaningful way in a lower-dimensional space.

Following processing by the improved t-SNE technique, the data is efficiently stored and retrieved by encoding it into QR codes. The reduced-dimensional data is contained in the QR codes, which facilitates easy access and sharing. To ensure compatibility and dependability, QR codes are generated from the processed data using tools or libraries.

For ease of access, the created QR codes and any related metadata are kept in a filesystem or database. Users can quickly retrieve the stored information by using mobile devices or scanners to scan the QR codes as part of a retrieval mechanism. After decoding the QR codes, this method extracts the relevant data for additional study or visualisation.

Iterative optimisation is used to adjust the parameters of the modified t-SNE method, feature extraction, and preprocessing during the process. This guarantees that the system's functionality is enhanced regularly, accommodating various medical picture kinds and analysis needs.

Ultimately, the suggested method's efficacy is confirmed by contrasting it with expert annotations or ground truth. To test the system's stability and generalizability across various settings and datasets, a wide range of medical pictures are used.

This technique describes a thorough method for effectively extracting, analysing, storing, and retrieving information from medical photos by utilising image processing, the modified t-SNE algorithm, and QR code technology.

From algorithm point of view, the final algorithm is as follows:



### High speed database design

In order to create a fast database that effectively retrieves data from QR codes, a number of technical approaches and techniques can be used. Using a distributed database design, which can manage massive data volumes and guarantee horizontal scalability, is one strategy. Furthermore, frequently accessed data is cached by using in-memory caching technologies like Redis or Memcached, which will decrease database latency.

Indexing and query optimization are important aspects of database optimization. In order to quickly get QR code information, it is possible to greatly improve query performance by creating the proper indexes on database fields. Multiple field or criterion queries can be supported effectively with composite indexes. Moreover, query performance can be further enhanced by putting query optimization strategies like cost-based optimization, query rewriting, and query planning and execution into practice.

To limit storage requirements and decrease the size of saved QR code data, data compression techniques like gzip or Snappy can be utilized. By lowering I/O overhead, columnar storage formats like Parquet or ORC can also be used to optimize data storage and enhance query performance. Data distribution and parallelism can be enhanced by partitioning the database tables according to access patterns or query needs. Fault tolerance and high availability are ensured via data replication over numerous nodes, allowing for quick retrieval even in the case of node failures.

To avoid overloading any single node, load balancing techniques divide incoming database queries evenly among several nodes. By minimizing the effort involved in creating and destroying connections, connection pooling effectively manages database connections. In high-speed database operations, robust concurrency control mechanisms—like optimistic concurrency control (OCC) or multi-version concurrency control (MVCC)—ensure data consistency and integrity. Effective transaction management strategies guarantee ACID characteristics and reduce transaction overhead.

Continuous database performance monitoring is made possible by the deployment of monitoring tools and performance indicators, which help locate bottlenecks and optimizer opportunities. The best possible database performance is ensured by routine performance tuning tasks including query optimization, index maintenance, and database configuration modifications based on workload trends and performance measurements. A high-speed database designed for effectively accessing data encoded in QR codes can be created by combining these design techniques and technical tactics, providing quick and dependable access to vital data in medical applications.

## Experimental results and discussions

The Q-DL approach uses a variety of cutting-edge procedures, including SVD and tSNE, to analyze images, retrieve information, compress images, diagnose problems, and save data. The whole strategy is evaluated using a range of metrics, including F1, Accuracy, and Recall. The outcomes are then contrasted with those of other approaches now in use, including QR-Blockchain [9], GAN [10], CNN [10], and image fusion technology [11].

It should be emphasized that the architecture was created with biomedical imaging in mind especially, hence existing datasets from online libraries were utilized to test the setup.

### Experimental setup

Python and R are used in this research to implement the suggested technique. The implementation makes use of pre-built packages [6].

Specifics of the experimental configuration and the packages utilized, as given in Table 2.

**Table 2:** Experimental specifications

#	Particular	Specification/Details
1	Processing Unit	I7-14700 K
2	Computing Memory	8 GB
3	CPU Clock Frequency	3.6 GHz
4	IDE (Python)	PyCharm
5	IDE (R)	R Studio
6	Packages (Python)	TensorFlow, Caffe
7	Packages (R)	TensorFlow, H2O

The performance of the proposed methodology is tested/implemented using hardware and software of the following specifications in Table 3.

**Table 3:** Hardware/Software specifications

#	Parameters	Specification/Details
1	Epochs per iteration	1000
2	Data batch size	300
3	Learning speed	0.001
4	Neural Net Activation Function	Sigmoid
5	Total Hidden Layers	50
6	No. of Neurons Per Layer	10
7	Drop Out Rate	0.1
8	Loss Function	MSE

The error function used in this study is Mean Squared Error (MSE), while the activation function is RELU.

With a bundle size of 300 and a dropout rate of 0.1, the learning rate is set at 0.001. A continuous time invariant probability distribution is taken into consideration for the use of modified t-SNE methodology with Bhattacharya Distance in order to improve the performance of the Q-DL approach.

### Dataset description

To apply the Q-DL in the article, radiographic and histology datasets are used from Crawford IDA [27] and Duke Radiology [28].

The 48,000 data points are gathered from references [8] and [9]. The Table 4 description is provided below.

**Table 4:** Description of dataset

#	Description	Detail
Data Source	[27] & [28]	Available online
No. of Data Points	30,000	-
No. of Columns	12	Images of X-Ray, CT Scan, Pictures, Date Time, Age, Sex, Region, etc

### Evaluation measures

Recall, accuracy, specificity, F1-score, and other assessment data are used to gauge how well the suggested approach performs. The following mathematical equations serve as the foundation for evaluating these measures' performance.

- Accuracy: This measure evaluates the model’s performance in correctly categorising data points pertaining to the severity of cyberbullying.
- Precision is a statistic that shows how consistently the model performs overall as well as how often it generates accurate classifications.
- Recall: This measure shows the number of positive values that are randomly evaluated based on the overall positive categorization input.
- The F1-score is a calculated value that may be thought of as the harmonic mean of the recall and accuracy functions.
- Specificity: Another really fundamental statistic, this one is basically the opposite of accuracy. This is the total number of negative hits in the model out of all the negative values.

### Performance analysis

The following two modalities have been used to assess the statistical performance of the suggested model for QR-based diagnosis from medical images and high-speed data retrieval:

1. A literature review comparison of the deep learning model to alternative approaches that are currently in use.
2. A comparison of the statistical outcomes of the deep learning model with the system's performance across different epoch counts.

**Fig. 4:**
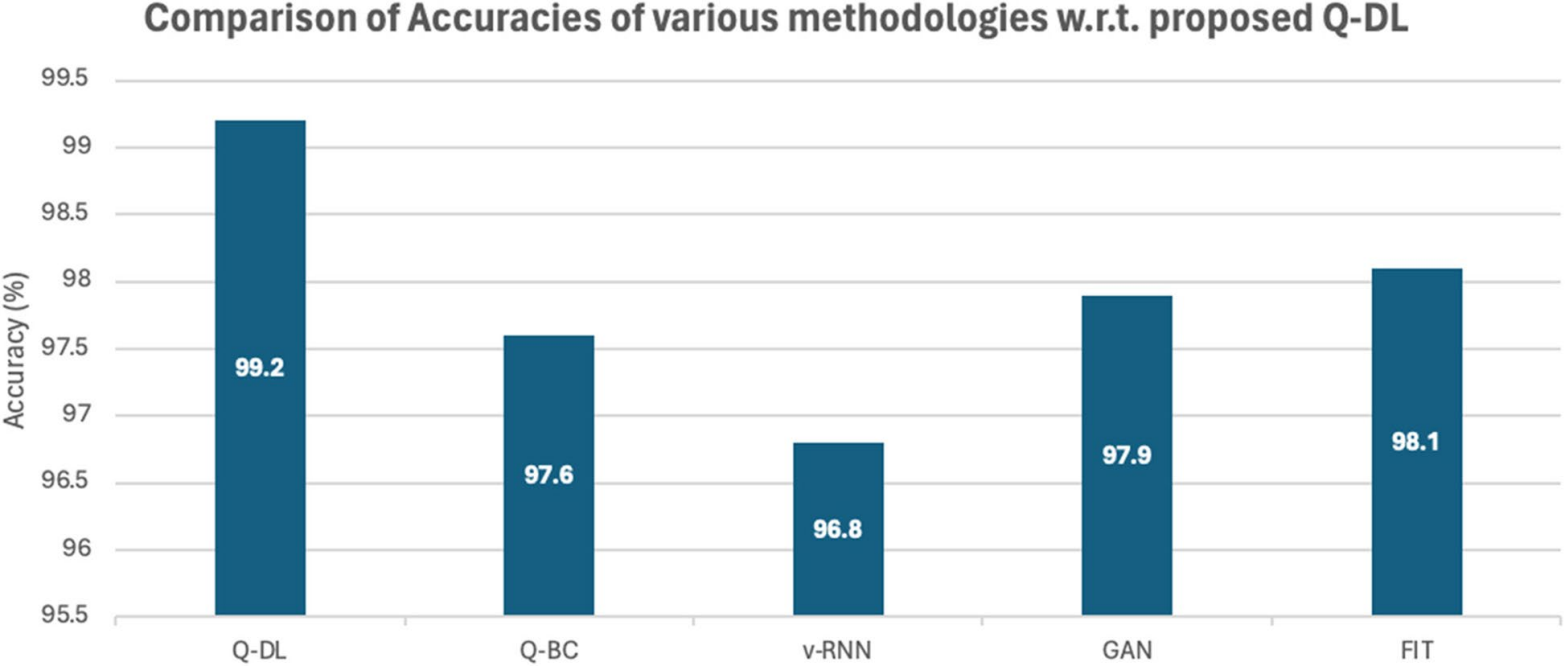
Comparison of accuracy

**Fig. 5:**
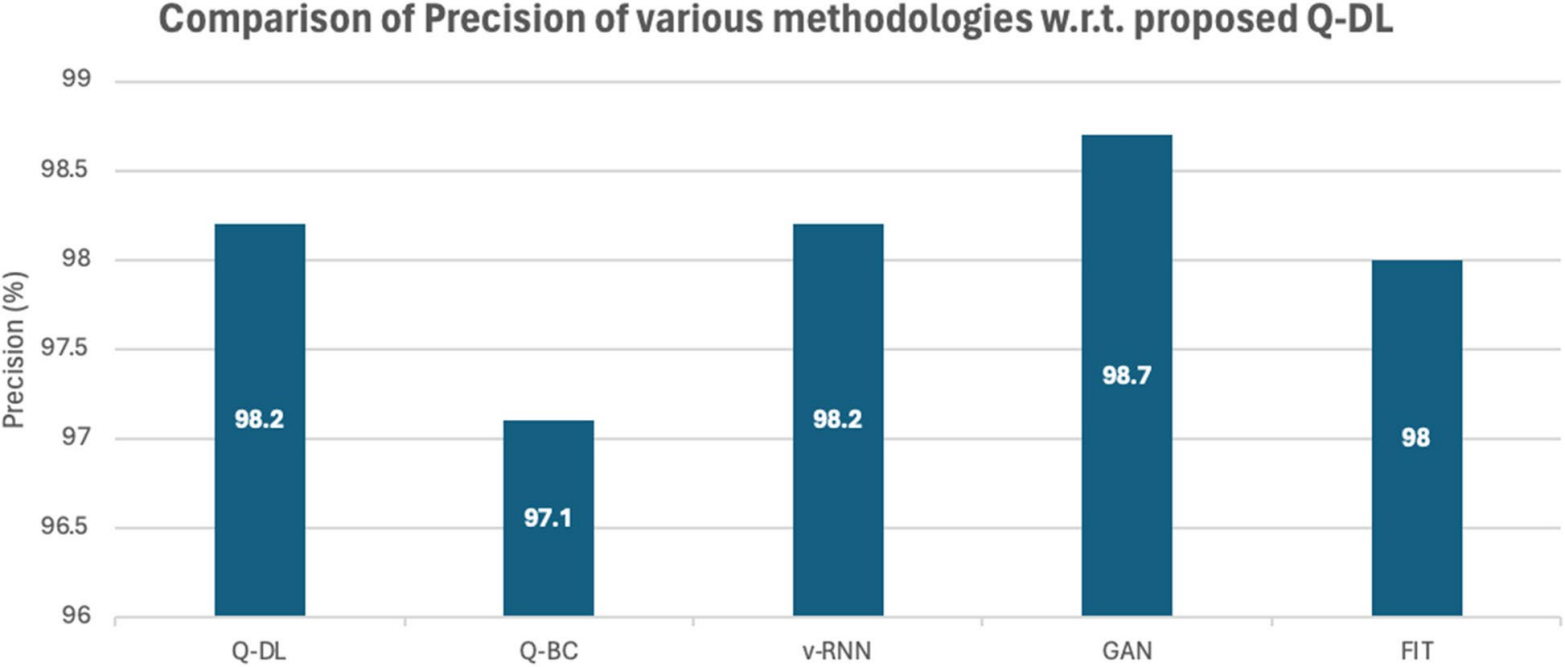
Comparison of precision

The Q-DL approach is assessed using a range of assessment metrics in comparison to other approaches, including Fusion Image Technology (FIT), Generative Adversarial Networks (GAN), QR-based blockchain (Q-BC), and Vanilla RNN (v-RNN) [27, 28]. Figures 4 and 5 below provide a visual representation of this unique approach Q-DL's performance in comparison to other existing methods like v-RNN, GAN, Q-BC, and FIT.

It is important to remember that the findings relate to the detection's overall accuracy.

Impressively, Q-DL method has managed to have a significant accuracy that is much higher than the available models from literature review, however, it is surprising to see that the Precision of the model is lower than GAN [20, 21]. This is happening, because in this model, similar data on the basis of similarity mapping is handled, therefore, some contextual information is lost in order to make the system run very fast.

However, since, a concept of hyperparameter tuning is not there in proposed model, therefore, precision plays a very small role in the overall working of the model.

**Fig. 6:**
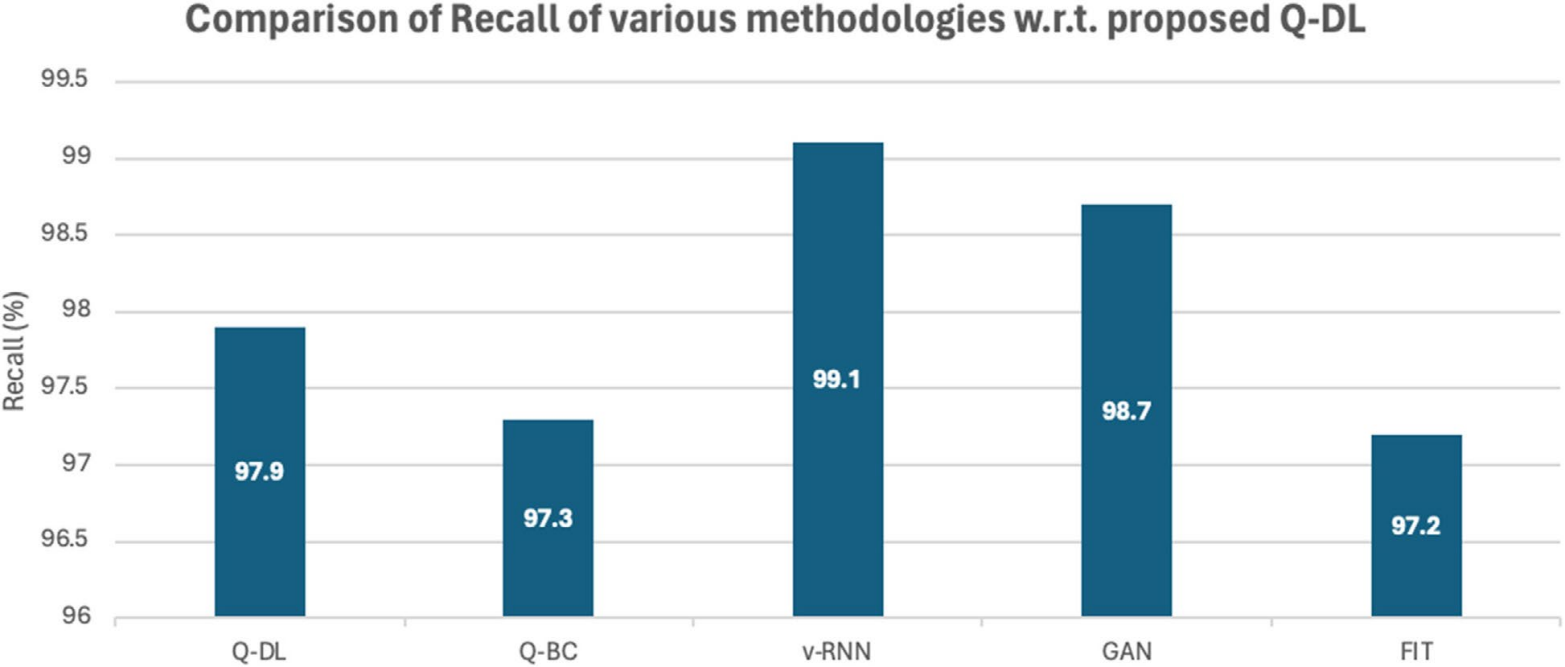
Comparison of recall measure

Reason for low precision of Q-DL model with respect to GAN is the low remembrance quality of Q-DL. Similarity mapping in Q-DL is used, this leads of overlapping or elimination of various features, which is otherwise held with GAN. Therefore, while running precision test, Q-DL doesn’t return significantly good results, when compared with GAN. The plot shown in Figs. 6 shows the statistical performance based on the accuracy and precision of the Q-DL method in comparison to other known models.

**Fig. 7:**
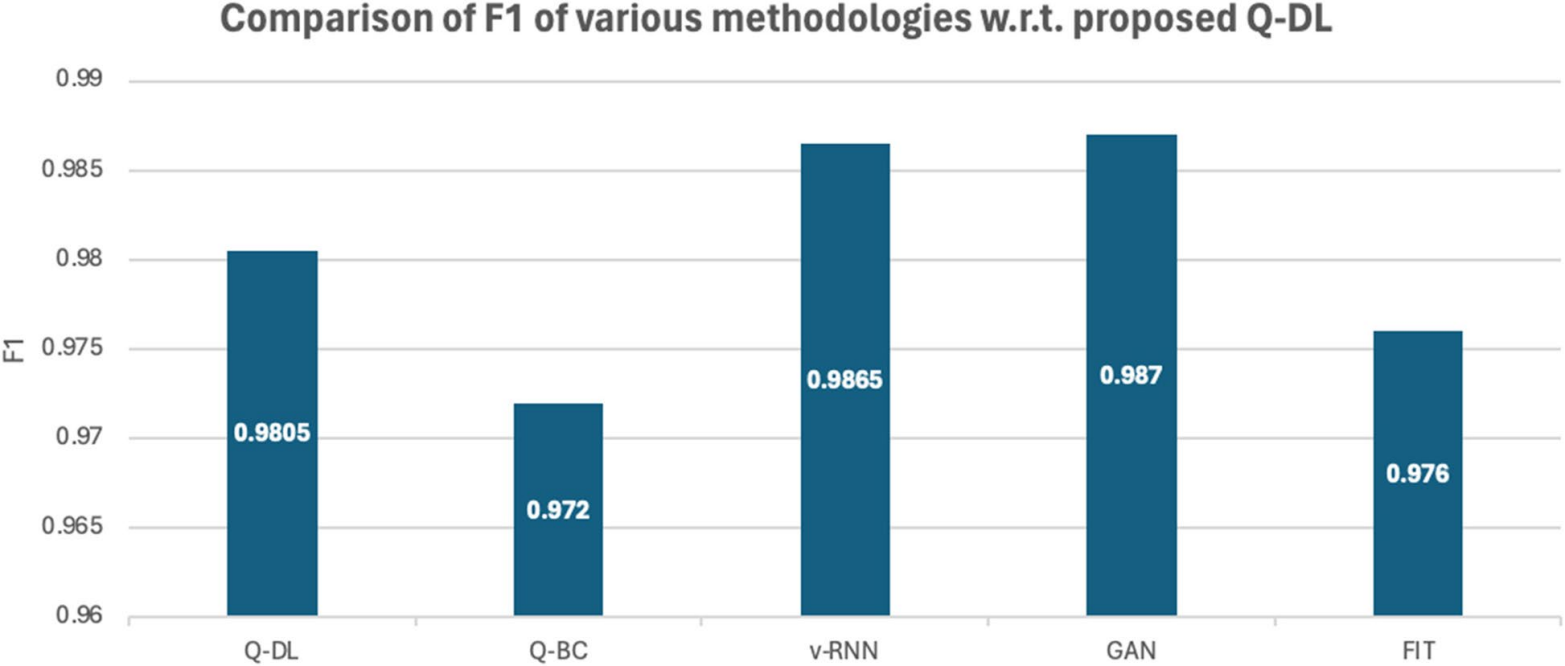
Comparison of F1-score

Similar to what is observed in the precision comparison, the recall of the model in Fig. 4 is also underperforming with respect to the other methodologies. The reason for this also same as that in the case of Precision, since a lot of information during similarity mapping is lost, a lot of contextual information is lost. However, there is nil impact on the performance of the system, since the model only does a preliminary mapping and does not give a complete diagnosis on its own. F score analysis is shown in Fig. 7.

Since the recall and precision performance of the proposed Q-DL method is worse than methods such as v-RNN and GAN, naturally, the F1 of Q-DL is giving an inferior output.

**Fig. 8:**
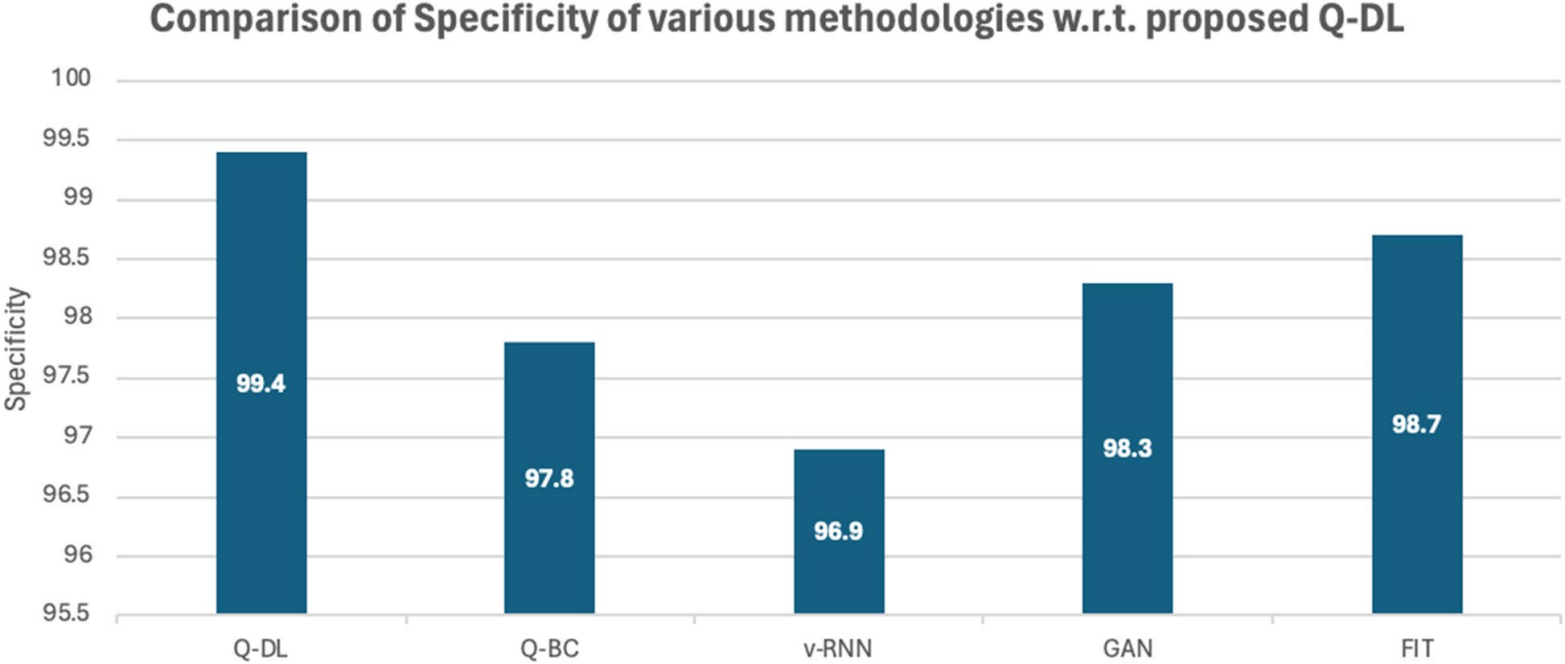
Graphical representation of specificity analysis

It is important to note that these metrics play no role in the overall working of the model. And this is a necessary tradeoff that is taken to make the system run at a faster rate [26]. The specificity analysis is shown in Fig. 8.

A comparative and cumulative result of the above computational studies has been given in the later parts of the paper. However, some noteworthy observations from the statistical analysis of the model are as follows:

1. The accuracy of Q-DL method is higher than most available methodologies.
2. The recall and precision of Q-DL is impacted due to loss of information from data compression and similarity mapping. However, it is to be noted that this tradeoff is necessary to achieve speed in the field, as chosen in the paper.
3. Specificity of the model is much higher than the other methodologies – this shows that in spite of some information being lost, the target features are still preserved.

**Fig. 9:**
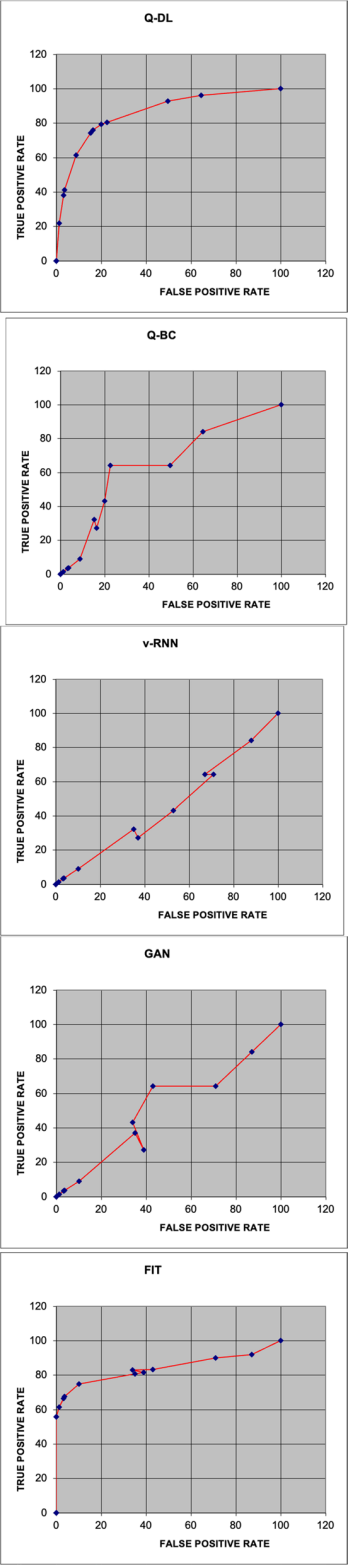
AUC-ROC plots

Now, the AUC-ROC plots of each methodology are plotted. The area under the curve for the suggested technique is displayed in the plot above in Fig. 9.

**Fig. 10:**
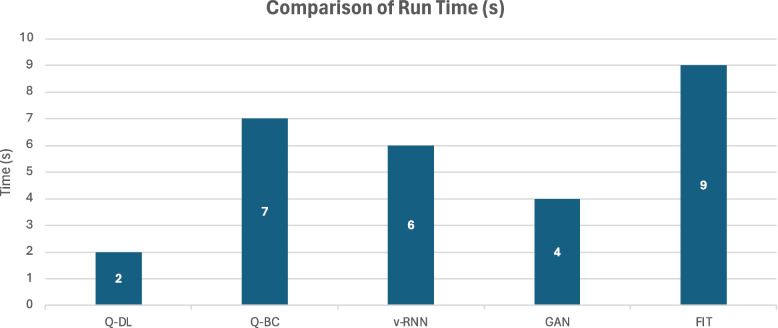
Comparison of run time

Comparing the suggested solution technique to the other models, the ROC value was higher at 0.9842. Figure 10 displays the models' run times.

ROC curve of GAN shows abrupt behavior due to the normalizations and matrix transformations (SVD) done on the base dataset for high-speed computations [29].

The statistical parameters of the suggested solution and the current models, including Q-BC, GAN, v-RNN, and FIT, have been compiled for comparison. The specifics of this analysis are provided in Table 5 below.

**Table 5:** Tabulation of statistical performance measure of various laid down processes against the proposed methodology

Model	Run Time(s)	Accuracy	Precision	Recall	F1	Specificity	AOC
Q-DL	2	99.2	98.2	97.9	0.9805	99.4	0.9842
Q-BC	7	97.6	97.1	97.3	0.972	97.8	0.9789
v-RNN	6	96.8	98.2	99.1	0.9865	96.9	0.9621
GAN	4	97.9	98.7	98.7	0.987	98.3	0.9655
FIT	9	98.1	98	97.2	0.976	98.7	0.9811

Not only was Q-DL compared to other Deep Learning models, but other implemented solutions' are also seen accuracy, which is shown in Table 6.

**Table 6:** Comparison of performance of various commonly known semi-supervised models when run on the base dataset

Method/Parameter	Accuracy	Recall	Precision	Specificity
Mean Teacher	87%	91%	96%	84%
t-SNE	96%	93%	95%	83%
Virtual Adversarial Training	92%	90%	91%	92%
Unsupervised Data Augmentation	91%	91%	86%	91%

The comparison of the previous techniques as part of validation is given in Table 7.

**Table 7:** Comparison of technique, dataset & accuracy of previous work done on the subject

#	Paper title & Ref No	Techniques Used	Dataset	Accuracy
1	**Wen et. al** [9]	**QR Codes + Blockchain**	**IDA**	**97.12%**
2	**Tao et. al ** [10]	**Image fusion technology**	**Github + Kaggle**	**97.34%**
3	**Tao et. al ** [10]	**Data breach issues**	**VISR Dataset**	**97.29%**
4	**Kumari et. al ** [11]	**Multi modal deep learning**	**IDA**	**96%**
5	**Proposed Q-DL methodology**	**Modified t-SNE for deep learning + SVD & Haar Cascades for image compression**	**Crawford & Duke**	**99.2%**

## Conclusion

In conclusion, the methodology proposed in the subject paper is a novel methodology towards a comprehensive approach for the efficient extraction, analysis, storage, and retrieval of information from medical images using advanced image processing techniques, a modified t-SNE algorithm, and QR code technology.

By leveraging image preprocessing methods and feature extraction techniques, successfully meaningful information is extracted from medical images, enhancing their quality and enabling the representation of key features. Through the application of a modified t-SNE algorithm, dimensionality reduction has been achieved while preserving essential data structure, facilitating visualization and analysis in a lower-dimensional space.

Furthermore, by encoding the reduced-dimensional data into QR codes [14], the storage is streamlined and retrieval process, allowing for fast and efficient access to critical medical information. The design of a high-speed database, utilizing distributed architecture, indexing, query optimization, data compression, and concurrency control, ensures rapid retrieval of QR code information, meeting the demands of real-time medical applications.

Through iterative optimization and validation, effectiveness and robustness of this approach is demonstrated, offering a novel solution for healthcare professionals to extract insights and make informed decisions based on medical imaging data. Moving forward, further research and development in this area hold the potential to revolutionize medical imaging practices, ultimately leading to improved patient care and outcomes. There are some well-defined limitations of the paper, which are high transactions occurring at a very high speed – requiring a very robust infrastructure to reduce failures and data misses. In addition to this, the complex deep learning models increase implementation complexity at larger data sets. Low precision therefore, precedence-based results shall lose its relevance in the same.

## Data Availability

The dataset used for the findings is publicly available and the source is well mentioned in the paper.
